# Books Reviewed

**Published:** 1868-07

**Authors:** 


### Books Reviewed.

Chambers on Indigestion. Second American from the second London edition. Philadelphia: Henry C. Lea, 1868.

Everybody has read the first edition of this work, and probably everybody will read the second and regard it as a great deal better than the first. It contains twelve chapters, the first being an introduction, in which the author has told us many things which could not have properly been presented under any other head. The second is upon the digestion of various kinds of food. Third, upon habits of social life leading to indigestion. We then have abdominal pain, vomiting, flatulence, diarrhœa, constipation and costiveness, nerve disorders connected with indigestion, analysis of cases, and alphabetical index completing the remainder of the book. This is a practical work upon indigestion. Cases are related showing nearly every phase of the disease and the treatment adopted, or that best calculated to restore the functions to natural action. This feature of clinical observation lends great attraction and adds value to the work; it brings the theoretical and practical in so close relationship that both are modified and improved. Dyspepsia includes and fathers a host of maladies, and Dr. Chambers has found them all and placed with them the proper remedies. It is a very good book, and every physician can read it with advantage.

Wilson on Diseases of the Skin, with plates and illustrations. Philadelphia: Henry C. Lea, 1868.

The early appearance of another edition of this work indicates the favor with which it is received by the profession. This seventh edition has received addition of the plates prepared by Mr. Wilson to illustrate his work on "Constitutional Syphilis and Syphilitic Eruptions." The recent editions of this work have treated the affections arising from syphilis somewhat extensively, making these plates illustrative of the disease very appropriate.

It is quite unnecessary to speak at all in detail concerning the contents or merits of this book; it is too well known and too highly appreciated by the profession to make such notice proper. No work upon this subject is better or more reliable, and no one has better arrangement or more thorough illustration.

Upon the subject of classification upon which so much has been written, the author in his preface says: "After much study of the principles of classification, we have succeeded in framing one, which, deriving its origin from the nature of the diseases themselves, will, we believe, after careful analysis, be found to be the most simple and the most practical that could be adopted. The suggestion of this arrangement arises from our experience at the bed-side of the patient; hence we have termed it *The Clinical Classification.*" The author goes on to show how he has followed up his plan, and the manner in which he has formed his various groups of diseases. We accept his classification as being as good as any and like the name he has given to it; it seems to us a very common-sense name, and that it is well bestowed.

Biddle's Materia Medica for the use of Students. Third edition, with illustrations. Philadelphia: Lindsay & Blakiston, 1868.

This is a condensed treatise upon the materia medica, designed to contain all that the student of medicine requires to learn, so arranged that he finds only what he really requires, freed from extraneous and unimportant matter. Several substances not introduced in previous editions have been added, viz: Calabar Bean, Woorara, Coca Guanara, Mate, Regoline, Bichloride of Methylene, Compounds of Amyl, Tertrachloride of Carbon, Nitrous Oxide, the Sulphites and Hydrosulphites, Carbolic Acid, Ammoniated Hydrogen, Iodide of Ammonium, Iodide of Sodium, and Iodoform. The hypodermic method of introducing medicine and the *atomization* or pulverization of fluids are treated at length. The work contains a succinct account of nearly all articles of the materia medica, and is eminently suited to the student while attending lectures and to the busy practitioner who desires to find the useful and thoroughly practical, with as little expenditure of time as possible. The author appears to have constantly in view the wants of medical students, and has to our mind, really conferred a great favor upon them in the preparation of the work. He very considerately and appropriately dedicates it to the gentlemen in attendance upon the various medical schools in the United States, and we earnestly and heartily recommend it to their attention and study.

---

Hewett on the Diseases of Women. First American from the second London edition, revised and enlarged, with one hundred and sixteen illustrations. Philadelphia: Lindsay & Blakiston, 1868.

This work is founded upon lectures delivered at St. Mary's Hospital School, and in its present edition is designed to form a complete treatise upon the diseases of women. The second edition differs from the first in its arrangement, as well as in addition to the text and of numerous valuable illustrations, many of them original with the author.

All the various topics belonging to this division of medicine receive attention by the author, who not only gives his own opinions and the grounds upon which they are based, but also the opinions of others upon all controverted or doubtful points, and the arguments and evidences by which they are sustained. This is the first American edition of the work, though the first London edition has been for a long time in the hands of the profession, its character well known, and its worth appreciated. We have been carefully reading its pages to learn if any new views were presented to the medical public. While we fail to find anything especially new or unheard of, yet we see that all the more recent advances in the knowledge of uterine diseases, are fully embodied in this work, and that it is presented in a most masterly manner. There is no pretension, no show of self, no undue claims to discovery or priority; the presentation of truth is plain, simple, well illustrated and convincing. We cannot but admire the general style of the author, and manner in which he has arranged his work. Many books have recently appeared upon this subject, but no physician should regard his library complete in the recent literature of uterine disease until Hewett on the Diseases of Women has been added.

The Neuroses of the Skin; their Pathology and Treatment. By Howard F. Damon, A. M., M. D. Philadelphia: J. B. Lippincott & Co.

In this monograph the author proposes a new classification of cutaneous affections depending either upon lesions to sensation, secretion, nutrition or structure, perversions in nutrition being regarded as the foundation of all of these. It is of lesions to sensibility to which this work is devoted, which lesions the author regards as dependent either upon the sensitive or vaso-motor nerves. The affections classed under hyperæsthesia or exalted sensibility are dermalgia, prurigo, urticaria and zoster, the symptoms, causes, pathological condition and treatment to each of these being exhaustively discussed. Anæsthesia or diminished sensibility, the author regards of less frequent occurrence than the former, the local varieties of this disease occurring principally in lepra and elephanteasis. At the close of the volume the history, etc., of twenty-six typical cases are recorded which greatly adds to its practical value.

---

United States Sanitary Commission Memoirs. Edited by Austin Flint, M. D.

This is the first of a series of volumes, intended by the United States Sanitary Commission to be published with a view "to lessen the evils of warfare as far as possible by a systematic and efficient employment of sanitary measures." Topics relating to the Causation and prevention of Disease, Camp Disease, and an elaborate Report upon the Diseases of the Federal Prisoners at Andersonville, Ga., being an official account of personal observations made by Prof. Joseph Jones, upon the authority of the Surgeon-General of the Confederate army, constitute the contents of the present volume. The contributions have been from men distinguished as careful observers and from such as had "opportunities for special studies in hospitals and in the field." The arrangement and selection of the monographs have been made by Prof. Austin Flint, which fact is a sufficient guarantee to the profession that its compilation is perfect. Upon all the points noticed it is highly instructive. The experience of the late war is to be carefully gathered, and when fully collected, will furnish a standard more valuable for comparison and more complete than has ever before been obtainable.

---

Institutes of Medicine. By Martyn Paine, A. M., M. D., LL. D. Eighth edition, revised. New York: Harper & Brothers, 1867.

The philosophy of medicine as presented by Prof. Paine in his well known work, is again brought to our notice in the appearance of the eighth edition, and we believe all true students of medicine will appreciate the fact that a great store-house of medical philosophy is thus again opened up to the profession. It would be pleasant and well worth the effort, had we space, to present the author's views upon a great many subjects, but it is wholly inconsistant under the circumstances to attempt anything of this sort. The work embraces a wide range of topics and deals in the occult, explaining as far as possible the obscure and undetermined, in connection with well demonstrated truth. The mature thought and reflection of a masterly mind, given to a long life of careful study is comprised in the work before us, and we cannot peruse its pages without being more and more impressed with the immense labor which has been bestowed upon it. It will richly repay the careful, reflective reader, and nothing but attentive study can afford any adequate idea of the chain of medical philosophy which has given the author his merited distinction.

